# The relationship between resilience and quality of life in advanced cancer survivors: multiple mediating effects of social support and spirituality

**DOI:** 10.3389/fpubh.2023.1207097

**Published:** 2023-08-28

**Authors:** Cancan Chen, Xiaofei Sun, Zhenya Liu, Miaorui Jiao, Wanhong Wei, Yanli Hu

**Affiliations:** ^1^Henan Provincial Key Medicine Laboratory of Nursing, Henan Provincial People’s Hospital, Zhengzhou University People’s Hospital, Zhengzhou, Henan, China; ^2^Department of Publicity, Zhengzhou Vocational University of Information and Technology, Zhengzhou, Henan, China; ^3^Department of Traditional Chinese Medicine, Affiliated Tumor Hospital of Zhengzhou University, Zhengzhou, Henan, China; ^4^School of Nursing and Rehabilitation, Zhengzhou University, Zhengzhou, Henan, China; ^5^School of Nursing, Guangzhou Medical University, Guangzhou, China

**Keywords:** cancer, quality of life, resilience, social support, spirituality

## Abstract

**Background:**

While previous studies have revealed a positive association between resilience and quality of life in advanced cancer survivors, the mechanisms of the relationship is still unclear. This study aimed to explore the relationships between resilience, social support, spirituality, and quality of life and determine the multiple mediation effects of social support and spirituality on the relationship between resilience and quality of life.

**Methods:**

With 286 advanced cancer survivors, a cross-sectional, correlational survey was adopted using convenience sampling. Resilience, social support, spirituality, and quality of life were evaluated by self-report questionnaires. The PROCESS macro for SPSS was used to test the multiple mediation model.

**Results:**

The scores for resilience, social support, spirituality and quality of life were positively correlated with one another. Resilience was found to be directly impact quality of life. Meanwhile, the relationship between resilience and quality of life was mediated by social support (effect = 0.067, 95% CI [0.019, 0.120]) and by spirituality (effect = 0.221, 95% CI [0.134, 0.332]), respectively, and by these two serially (effect = 0.036, 95% CI [0.015, 0.067]).

**Conclusion:**

Social support and spirituality played multiple mediating roles in the relationship between resilience and quality of life. Interventions aimed at increasing resilience, and then boosting social support and spirituality may be beneficial for promoting quality of life of advanced cancer survivors.

## 1. Introduction

Cancer is a severe public health issue and a significant barrier to increased life expectancy worldwide (1). In China, it ranks as the leading cause of death, with approximately 4 million new diagnoses and 3 million deaths in 2020 (1). A cancer diagnosis may be followed by symptoms including persistent pain, limited functioning, and emotional and physical trauma secondary to the prescribed therapies and disease progression, all of which have a negative influence on physical, psychological, and social elements of quality of life (2). Quality of life is a multidimensional concept that focuses on patients’ perception of their own health status as well as nonmedical aspects of their lives (3). It is acknowledged as a valuable indicator of patient-reported outcomes and is critical for assessing the overall therapeutic benefit and functional rehabilitation of patients during the course of their lives (2). As a result, healthcare providers must promote quality of life in survivors of advanced cancer; identifying modifiable factors might be the first step.

Resilience is described as the capacity of an individual to adapt in the face of tragedy, trauma, hardship, and ongoing significant life stressors to maintain normal physiological and psychological functions (4). Cancer is accepted as an enormous stressor that triggers adverse emotions, such as anxiety, depression, and fear, severely affecting quality of life (2). The Neuman Systems Model (NSM) is a holistic system perspective that focuses on the stressors that may harm to a person’s well-being as well as mechanisms that may reduce the impact of stress altogether. The NSM provides a framework that can be used to explore interactions between persons and their environment and how individuals respond to stressors in the environment (5). To interact with the environment, the client system develops a set of defenses (e.g., physical and psychological defenses) that provide protection against a variety of stressors. Stress, defense, and nursing interventions are the major components of NSM. When a stressor operates on the person, the body will make a response defensively to prevent stressors from invading the normal system. The goal of nursing intervention is to maintain and restore the balance of the client system. Each cancer survivor is an independent system; the disease and its treatment-related factors (stressors) will have a variety of adverse impacts on patients (system), which in turn can lead to changes in their psychological toughness, such as resilience (body defense). The active individual’s responses to body defenses influence patient-reported outcomes (6). Prior studies have confirmed that strong resilience was associated with better quality of life in advanced cancer survivors, which is advantageous to one’s health (6, 7). However, the evidence for the pathways in the link between resilience and quality of life in advanced cancer survivors is limited.

Social support refers to the various types of assistance from a social network (e.g., family members, friends, and significant others), which may be formal and/or informal, including emotional, instrumental, and informational support (8). Previous studies have indicated that patients with high resilience tend to perceive a high level of social support (6, 9). Moreover, social support, as a positive source, helps cancer survivors ease psychological distress and promote their quality of life (10, 11). As a result, we hypothesized that social support would be a potential mediator between resilience and quality of life in advanced cancer survivors.

A life-threatening disease diagnosis, such as cancer, may trigger suffering and existential distress (12). Spirituality is an integral part of health (13). However, spirituality has no universal definition and is indeed influenced by culture (14, 15) and expressed in various ways according to culture or, to some extent, religion (16). In China, however, people are less Christian-orientated than those in Western countries (17). Spirituality, in the context of China, reflects an emphasis on individual inner peace through connection and harmony with oneself, others, and nature or a higher power (heavenly principles). Furthermore, the spiritual categories of the Chinese mostly concern intra-, inter-, and transpersonal connectedness and transcendence (18). Spirituality is described as the aspect of humanity that refers to how individuals seek and express meaning and purpose, as well as how they sense their connectedness to themselves, others, nature, and transcendence, such as a sense of meaning in one’s life, harmony, peacefulness, and a sense of strength and comfort from one’s faith (19). Studies have demonstrated that spirituality can assist cancer survivors in adjusting their perceptions of unpleasant symptoms and traumatic events, making them more tolerable and improving their quality of life (20, 21). There is evidence that resilience positively correlates with spirituality in psoriasis (22), hemodialysis (23), and breast cancer survivors (24). Furthermore, spirituality, a component of life, can reduce the symptoms of illness and bring hope, comfort, and value to one’s experience (25), in turn improving individuals’ quality of life (12, 26). Therefore, we hypothesized that spirituality would serve as a mediator in the association between resilience and quality of life. In addition, the link between social support and spirituality in cancer survivors was tested. Ciria-Suarez et al. (27) and Li et al. (28) found that social support had a positive relationship with spirituality. Moreover, social support can promote spirituality and life transition adaptation (27). We thus hypothesized that social support and spirituality would play serially mediating roles in the relationship between resilience and quality of life.

**Figure 1:**
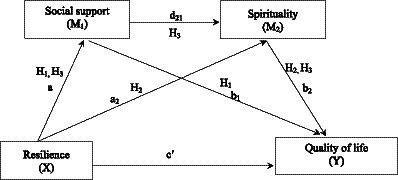
The multiple mediation model.

Following the NSM, we operationalized resilience as a body defense and social support and spirituality as individual’s responses, with quality of life as the patient-reported outcome. The purpose of this study was to test the multiple mediating effects of social support and spirituality on the link between resilience and quality of life in advanced cancer survivors. Based on the above mentioned contents, this study proposes the following hypotheses (Figure 1). First, social support mediates the association between resilience and quality of life. Second, spirituality mediates the association between resilience and quality of life. Finally, social support and spirituality would have a serial mediating effect between resilience and quality of life in advanced cancer survivors. The findings of this study will offer new evidence for developing multimodal intervention programs that can enhance the quality of life of advanced cancer survivors.

## 2. Materials and methods

### 2.1. Recruitment and participants

In our cross-sectional, correlational design, we recruited cancer survivors using convenience sampling from one hospital in China from June 2018 to July 2019. Patients were approached by a trained researcher, who explained the purpose of the study. Prior to the survey, informed consent was acquired from all participants who met the eligibility criteria. The questionnaires were filled out and collected on the spot. The eligibility criteria of participants were as follows: (1) 18 years old or above; (2) clinically diagnosed with cancer; and (3) classified as Tumor Node Metastasis (TNM) stage III-IV. Subjects with cognitive function and mental disorders or auditory or visual impairments were excluded. This study was approved by the Ethics Committee of Affiliated Tumor Hospital of Zhengzhou University (number: 2019014) and registered as a trial with the Chinese Association of Clinical Practitioners (number: ChiCTR1900020930).

The sample size was calculated using G*Power Version 3.1 and linear multiple regression, with the specifications fixed model, deviation of *R*^2^ from zero, in the “F tests” family was run (29), with a moderate effect size (*f*^2^ = 0.15) (30), alpha of 0.05, and power of 0.90. The number of participants computed was 136. Considering a 20% dropout rate (31), including refusal to participate in interviews and failure to finish the survey, we required 164 participants. Thus, 286 participants were considered sufficient. Of the 286 survivors with advanced cancer in the current study, more than half were under the age of 60 (66.8%), were male (63.3%), had an education level below high school (61.5%), and were employed (66.8%). Most participants were married or cohabiting (91.3%), had no religious beliefs (83.6%), and earned less than 3,000 RMB per month (82.2%). In addition, almost half of the participants had suffered cancer for 6 months (44.1%). The details are presented in Table 1.

**Table 1:** Characteristics of the sample (*N* = 286).

Variable	Categories	*n* (%)
Age	<60 years	191 (66.8)
	≥60 years	95 (33.2)
Gender	Male	181 (63.3)
	Female	105 (36.7)
Education	<High school	176 (61.5)
	≥ High school	110 (38.5)
Marital status	Single/divorced/widow	25 (8.7)
	Cohabiting/married	261 (91.3)
Employment	Unemployed	95 (33.2)
	Employed	191 (66.8)
Religion	No	239 (83.6)
	Yes	47 (16.4)
Household monthly income	<3,000 RMB	235 (82.2)
	≥3,000 RMB	51 (17.8)
Disease duration	<6 months	160 (55.9)
	≥6 months	126 (44.1)

### 2.2. Measures

#### 2.2.1. Demographic and clinical characteristics

Data related to demographic and clinical characteristics were obtained using an investigator-developed general information questionnaire. The variables in this study included age, gender, education, marital status, employment, religion, household monthly income, and disease duration. The demographic and clinical data were obtained from medical records and patient interviews.

#### 2.2.2. Resilience

Resilience was measured using the 10-item Chinese version of the Connor-Davidson Resilience Scale (CD-RISC10) (32, 33). The scale contains 10 items (e.g., “I believe I can achieve my goals, even if there are obstacles”). Answers are scored from 0 to 4 (0 being “not true at all” and 4 “being almost always”). The sum of item means is used as the final score on the scale. Total scores vary from 0 to 40, with a higher number reflecting greater resilience. The CD-RISC10 has demonstrated high internal consistency (33). Cronbach’s alpha was 0.947 in the present study.

#### 2.2.3. Social support

The Chinese version of the Multidimensional Scale of Perceived Social Support (MSPSS) was used to assess perceived social support (34, 35). The scale comprises twelve items, which are divided into three dimensions: family support (e.g., “My family really tries to help me”), friends’ support (e.g., “I can talk about my problems with my friends”), and significant others’ support (e.g., “There is a special person who is around when I am in need”). Each question is graded on a seven-point Likert scale, ranging from 1 (“strongly disagree”) to 7 (“strongly agree”). The sum of item means is used as the final score, ranging from 12 to 84. A higher score suggests a higher level of social support. The Chinese MSPSS has been well validated in patients (35). The Cronbach’s alpha for the MSPSS was 0.943 in the current study.

#### 2.2.4. Spirituality

The Chinese Functional Assessment of Chronic Illness Therapy-Spiritual Scale (FACIT-Sp-12) (36), which was part of the Functional Assessment of Chronic Illness Therapy Scale (FACIT) (37), was used to evaluate spirituality. The FACIT-Sp-12 consists of 12 items covering three dimensions: belief (e.g., “I find strength in my faith or spiritual beliefs”), meaning (e.g., “I feel a sense of purpose in my life”), and peace (e.g., “I feel peaceful”). All questions in the scale use a five-point Likert scale, with 0 being “not at all” and 4 being “very much.” The sum of item means of the scale is used. The total score ranges from 0 to 48, with higher scores indicating greater spirituality. Cronbach’s alpha was 0.871 in the current study.

#### 2.2.5. Quality of life

The Chinese Functional Assessment of Cancer Therapy-General scale (FACT-G), one of the most widely used quality of life assessment instruments in cancer research, was used to measure quality of life (38, 39). The FACT-G measures common components of quality of life in cancer survivors. The scale is composed of 27 items covering four dimensions: physical (e.g., “I feel nauseous”), social and familial (e.g., “I can get the emotional support from my family”), emotional (e.g., “I feel nervous”), and functional well-being (e.g., “I can enjoy my life”). Each response ranges from 0 (“not at all”) to 4 (“very much”) on a 5-point Likert scale. The sum of item means is used. The total scores vary from 0 to 108, with higher scores suggesting better overall quality of life. Cronbach’s alpha was 0.861 in the present study.

### 2.3. Statistical analysis

Statistical analyses were performed with IBM SPSS Statistics version 26.0 (IBM Corp, Armonk, NY, United States). Continuous data are represented by the mean and standard deviation (SD), whereas categorical data are summarized by frequency and percentage. The Pearson correlation coefficient was used to examine the correlations between resilience, social support, spirituality, and quality of life. Linear regressions were used to examine the relationship among resilience, social support, spirituality, and quality of life, adjusting for demographic and clinical variables (i.e., age, gender, education, marital status, employment, religion, household monthly income, and disease duration). Model 1 included covariates; Model 2 included covariates and resilience; and Model 3 included covariates, resilience, social support, and spirituality.

We adopted Model 6 of the PROCESS macro for SPSS developed by Hayes to analyze the multiple mediation model (40). In this model, X influences Y via four paths, containing a direct influence and three indirect effects. Resilience was set as X, social support as M_1_, spirituality as M_2_, and quality of life as Y in this study. The indirect effects were: (i) via social support (a_1_b_1_); (ii) via spirituality (a_2_b_2_); and (iii) via social support and spirituality in serial (a_1_d_21_b_2_). The direct effect of X on Y was represented by coefficient c’. Total effects (c) are made up of direct and total indirect effects, derived as follows: c = c’ + a_1_b_1_ + a_2_b_2_ + a_1_d_21_b_2_. We standardized all variables (Z values) before performing mediation analyses. The point estimates and 95% confidence interval (CI) of direct effects, indirect effects, and total effects were examined using bootstrapping with 5,000 simulations. If zero was excluded from the 95% CI interval, the effect was statistically significant.

## 3. Results

### 3.1. Correlations of resilience, social support, spirituality and quality of life

The scores and correlations of resilience, social support, spirituality and quality of life are displayed in Table 2. The scores for resilience, social support, spirituality and quality of life were 32.35 ± 9.64, 62.35 ± 13.86, 36.13 ± 10.37, and 69.86 ± 16.65, respectively. The results of the Pearson correlation indicated that resilience was positively correlated with social support (*r* = 0.507, *p* < 0.01), spirituality (*r* = 0.626, *p* < 0.01), and quality of life (*r* = 0.591, *p* < 0.01). In addition, scores for these four variables positively correlated with one another.

**Table 2:** Scores and correlation coefficients of the study variables (*N* = 286).

Variable	Mean ± SD	1	2	3	4
1.Resilience	32.35 ± 9.64	1	–	–	–
2.Social support	62.35 ± 13.86	0.507^**^	1	–	–
3.Spirituality	36.13 ± 10.37	0.626^**^	0.446^**^	1	–
4.Quality of life	69.86 ± 16.65	0.591^**^	0.451^**^	0.636^**^	1

SD, standard deviation.

** *p* < 0.01.

### 3.2. Linear regression analysis of quality of life

To identify factors associated with quality of life, linear regression was utilized (Table 3). None of the variables in this investigation exhibited multicollinearity issues. Resilience was found to be positively linked with quality of life in Model 2 (β = 0.596, *p* < 0.001) after controlling for demographic and clinical factors. The influence of resilience on quality of life decreased (β = 0.263, *p* < 0.001) when social support (β = 0.129, *p* < 0.05) and spirituality (β = 0.413, *p* < 0.001) were placed into Model 3, suggesting that social support and spirituality may be mediators of the relationship between resilience and quality of life.

**Table 3:** Linear regression analysis of quality of life (*N* = 286).

	Model 1			Model 2			Model 3		
	β	*t*	*p*	β	*t*	*p*	β	*t*	*p*
Constant		12.411	<0.001		7.523	<0.001		4.725	<0.001
Age	−0.037	−0.604	0.547	−0.045	−0.894	0.372	−0.047	−1.050	0.294
Gender	0.004	0.062	0.950	0.002	0.035	0.972	−0.030	−0.668	0.505
Education level	0.096	1.495	0.136	−0.032	−0.596	0.552	−0.011	−0.218	0.828
Marital status	0.049	0.821	0.412	−0.006	−0.125	0.900	0.001	0.024	0.981
Employment	0.108	1.749	0.081	0.072	1.432	0.153	0.051	1.118	0.265
Religion	−0.101	−1.668	0.096	−0.009	−0.186	0.853	−0.028	−0.624	0.533
Household monthly income	−0.002	−0.038	0.970	0.021	0.408	0.684	0.018	0.395	0.693
Disease duration	0.019	0.313	0.754	−0.022	−0.445	0.657	−0.023	−0.511	0.610
Resilience				0.596	11.871	<0.001	0.263	4.308	<0.001
Social support							0.129	2.470	0.014
Spirituality							0.413	7.179	<0.001
*R*^2^	0.031			0.358			0.485		
Adjusted *R*^2^	0.003			0.338			0.464		

β, standardized beta.

### 3.3. Multiple mediating effects of social support and spirituality

**Figure 2:**
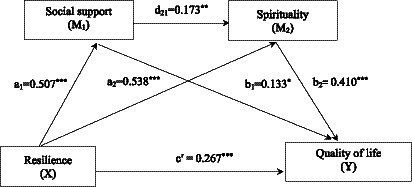
The multiple mediation model of social support and spirituality linking resilience and quality of life in advanced cancer survivors. ^*^p < 0.05, ^**^p < 0.01, ^***^p < 0.001.

The multiple mediation analysis results of social support and spirituality on resilience and quality of life are shown in Figure 2 and Table 4. The total effect (effect = 0.591, 95% CI [0.497, 0.685]) and total indirect effect (effect = 0.324, 95% CI [0.223, 0.436]) of resilience on quality of life were significant. Resilience had an indirect effect on quality of life via social support (effect = 0.067, 95% CI [0.019, 0.120]) and spirituality (effect = 0.221, 95% CI [0.134, 0.332]), accounting for 20.6 and 68.2% of the total indirect effect, respectively. At the same time, resilience had an indirect effect on quality of life via social support and spirituality in serial (effect = 0.036, 95% CI [0.015, 0.067]), accounting for 11.1% of the total indirect effect. Furthermore, the direct effect of resilience on quality of life was statistically significant (effect = 0.267, 95% CI [0.152, 0.382]).

**Table 4:** Mediation analysis of resilience and quality of life (*N* = 286).

	Effect	SE	LLCI	ULCI
Total effect	0.591	0.048	0.497	0.685
Direct effect	0.267	0.058	0.152	0.382
Total Indirect effect	0.324	0.055	0.223	0.436
Resilience → social support → quality of life	0.067	0.026	0.019	0.120
Resilience → spirituality → quality of life	0.221	0.050	0.134	0.332
Resilience →social support → spirituality → quality of life	0.036	0.013	0.015	0.067

SE, standard error; LLCI, lower limit confidence interval; ULCI, upper limit confidence interval.

## 4. Discussion

To the best of our knowledge, this is the first study to demonstrate the multiple mediating effects of social support and spirituality on the link between resilience and quality of life in advanced cancer survivors. The results of this study revealed that resilience was positively associated with quality of life via three indirect pathways: (1) a relationship mediated by social support; (2) a relationship mediated by spirituality; and (3) a relationship serially mediated by social support and spirituality. The findings supported the NSM by demonstrating that patients with stronger resilience tended to perceive sufficient social support and a higher level of spirituality, which was related to better quality of life.

The current study revealed that social support mediated the association between resilience and quality of life in advanced cancer survivors. That is, patients who reported higher resilience perceived more social support, which in turn improved quality of life. Thus, hypothesis 1 was supported. Our results are similar to Zhou et al. (6) and Zhang et al.’s (41) studies that social support acted as a mediator in the relationship between resilience and quality of life in breast cancer patients. A systematic review showed that resilience was positively linked to social support in colorectal cancer survivors (9). A meta-analysis has confirmed that cognitive-behavioral therapy and mindfulness-based therapy can improve individual’s resilience (42). Social support systems are crucial protective elements for those going through stressful situations (43). Meanwhile, adequate social support is critical for helping cancer survivors manage their illness to maintain better quality of life (44). Our findings suggest that interventions aimed at enhancing resilience may promote social support, and consequently were associated with an improvement in quality of life.

The current study found that spirituality mediated the relationship between resilience and quality of life in advanced cancer survivors. Resilience can not only directly contribute to the quality of life of advanced cancer survivors but also effectively improve the quality of life by increasing their spirituality. Thus, hypothesis 2 was supported. In a systematic review, the existing evidence revealed that resilience was associated with quality of life in patients with advanced cancer (45). Spirituality can create meaningfulness and purpose, which in turn contribute to patients’ quality of life (12, 46). Despite the absence of conclusive evidence of a mediation effect, the findings in prior research confirmed our reports that spirituality at least partially explained the association between resilience and quality of life in advanced cancer survivors. Our findings emphasize the value of spirituality, particularly for advanced cancer survivors with low resilience. Previous studies have indicated that interventions aimed at promoting spirituality, such as life review interventions (47) and meaning-centered group psychotherapy (48) may be beneficial for improving quality of life in advanced cancer survivors.

The results of the present study further showed that social support and spirituality were serial mediators of the relationship between resilience and quality of life, which confirmed hypothesis 3. Patients with better resilience perceived higher levels of social support, followed by higher levels of spirituality, and hence improved quality of life. A 10-year follow-up study of cancer survivors found that greater social support perceived by cancer survivors was related to an increase in the probability of a mental state in steady-high trajectories and a decrease in the probability of a mental state in steady-low trajectories (49). Moreover, people with a diagnosis of cancer could rely on spirituality to help them cope with the illness and its treatment, which was correlated with better physical, mental and social quality of life (46, 50). According to the NSM, we established a framework describing the relationship between resilience (psychological defense), social support and spirituality (personal response), and quality of life (patient-reported outcomes). The findings further indicate that, aside from the direct influence of resilience on quality of life, strong resilience was related to a positive personal response (e.g., social support and spirituality) and hence a better patient-reported outcome (e.g., quality of life). Thus, developing multimodal intervention strategies should integrate social support and spirituality to enhance the favorable effect of resilience on quality of life in advanced cancer survivors.

This study has several limitations. First, the cross-sectional research design in our study could not determine the causal relationship between variables. Therefore, a longitudinal or interventional studies in the future are necessary. Second, the participants were recruited from a single tertiary hospital, which limited the generalizability of the findings. Multicenter studies should be conducted in the future to increase the representativeness of the results. Third, the sample shows quite low socioeconomic status in our study (SES; relatively low educational level and income, although low income is not surprising for such a vulnerable sample). Low SES may be associated with low resilience and quality of life. Therefore, our study should be generalized to other population cautiously. Finally, the self-reported measures used in our study may lead to response bias, despite the good reliability and validity of the instruments in preceding samples. Nonetheless, this study adopts a theoretical exploratory method to offer fresh perspectives on the relationship between resilience and quality of life in advanced cancer survivors.

## 5. Conclusion

Social support and spirituality were multiple mediators of the relationship between resilience and quality of life in advanced cancer survivors. Patients who experience stronger resilience may perceive higher levels of social support and are more likely to have higher spirituality, leading to better quality of life. As a result, it is necessary for healthcare providers to develop interventions that focus on boosting survivors’ resilience, and then increasing social support and spirituality to promote quality of life.

## Data availability statement

The original contributions presented in the study are included in the article/supplementary material, further inquiries can be directed to the corresponding authors.

## Ethics statement

This study was approved by the Ethics Committee of Affiliated Tumor Hospital of Zhengzhou University (number: 2019014) and registered as a trial with the Chinese Association of Clinical Practitioners (number: ChiCTR1900020930).

## Author contributions

CC was involved in conceptualization, formal analysis, and writing-original draft. XS was responsible in data acquisition and analysis. ZL and MJ participated in analysis and interpretation of data. WW and YH made equal contribution in substantive intellectual contributions to the conception of the work and the interpretation of the data and revised the manuscript. All authors contributed to the article and approved the submitted version.

## Funding

This work was supported by the Henan Provincial Medical Science and Technology Research Plan (joint construction; no. LHGJ20190654).

## Conflict of interest

The authors declare that the research was conducted in the absence of any commercial or financial relationships that could be construed as a potential conflict of interest.

## Publisher’s note

All claims expressed in this article are solely those of the authors and do not necessarily represent those of their affiliated organizations, or those of the publisher, the editors and the reviewers. Any product that may be evaluated in this article, or claim that may be made by its manufacturer, is not guaranteed or endorsed by the publisher.
